# Physicochemical Characteristics of Four Limonene-Based Nanoemulsions and Their Larvicidal Properties against Two Mosquito Species, *Aedes albopictus* and *Culex pipiens molestus*

**DOI:** 10.3390/insects11110740

**Published:** 2020-10-28

**Authors:** Ioanna Theochari, Athanasios Giatropoulos, Vassiliki Papadimitriou, Vasileios Karras, Georgios Balatsos, Dimitrios Papachristos, Antonios Michaelakis

**Affiliations:** 1 Institute of Chemical Biology, National Hellenic Research Foundation, 11635 Athens, Greece; jtheohari@eie.gr (I.T.); vpapa@eie.gr (V.P.); 2 Laboratory of Efficacy Assessment of Pesticides, Scientific Directorate of Pesticide’s Assessment and Phytopharmacy, Benaki Phytopathological Institute, 14561 Kifissia, Greece; a.giatropoulos@bpi.gr; 3 Scientific Directorate of Entomology and Agricultural Zoology, Benaki Phytopathological Institute, 14561 Kifissia, Greece; v.karras@bpi.gr (V.K.); g.balatsos@bpi.gr (G.B.); d.papachristos@bpi.gr (D.P.)

**Keywords:** nanoemulsions, limonene, electron paramagnetic resonance (EPR) spectroscopy, dynamic light scattering (DLS), larvicides, mosquitoes, *Aedes albopictus*, *Culex pipiens molestus*

## Abstract

**Simple Summary:**

The repeated use of synthetic insecticides against mosquitoes has posed negative impacts on the environment; hence, the utilization of more ecofriendly mosquito control products is deemed necessary. Nanoformulations of phytochemical substances with a safer profile for the environment have been considered potential larvicidal agents against mosquitoes. In this perspective, we formulated 4 (R)-(+)-limonene based oil-in-water nanoemulsions by low and high energy methods, and we studied their physicochemical characteristics (e.g., viscosity, stability, mean droplet diameter, polydispersity index). In addition, we evaluated their larvicidal properties against two mosquito species of great medical importance, *Culex pipiens molestus* and *Aedes albopictus.* Laboratory bioassays showed that limonene-based nanoemulsions either improved or did not affected limonene toxicity, depending on the delivery system and mosquito species. In conclusion, all tested limonene-based nanoemulsions provided sufficient toxic effects against mosquito larvae, serving as potential formulations for mosquito control applications.

**Abstract:**

Negative impacts on the environment from the continuous use of synthetic insecticides against mosquitoes has driven research towards more ecofriendly products. Phytochemicals, classified as low-risk substances, have been recognized as potential larvicides of mosquitoes; however, problems related to water solubility and stability are limiting factors for their use in mosquito control programs in the field. In this context, many researchers have focused on formulating essential oils in nanoemulsions, exploiting innovative nanotechnology. In the current study, we prepared 4 (R)-(+)-limonene oil-in-water nanoemulsions using low and high energy methods, and we evaluated their physicochemical characteristics (e.g., viscosity, stability, mean droplet diameter, polydispersity index) and their bioactivity against larvae of two mosquito species of great medical importance, namely, *Cx. pipiens molestus* and *Ae. albopictus.* According to the dose–response bioassays with the limonene-based nanoemulsions and pure limonene (dissolved in organic solvent), the tested nanoformulations improved the activity of limonene against *Ae. albopictus* larvae, while the performance of limonene was either the same or better than limonene against *Cx. pipiens molestus*, depending on the applied system. Overall, we achieved the production of limonene-based delivery nanosystems, with sufficient lethal properties against mosquito larvae to consider them promising larvicidal formulations applicable to mosquito breeding sites.

## 1. Introduction

During the past few years, there has been a growing interest in the formulation of nontoxic, biocompatible, and safe liquid nanosized systems, including nanoemulsions, nanoliposomes, solid lipid nanoparticles, polymer nanoparticles, dendrimers and nanosuspensions, for their potential technological applications in the food, cosmetic, pharmaceutical and agrochemical sectors [1,2,3,4,5].

Nanoemulsions are colloidal dispersions of liquid droplets in another nonmiscible continuous liquid phase, generally in the size range of 20–200 nm. They have unique properties, such as long-term stability, large surface area per unit volume and tuneable rheology [6,7]. Due to these characteristics, nanoemulsions are considered promising delivery systems for bioactive substances, especially those exhibiting low water solubility [8,9,10,11]. Nanoemulsions can be prepared by either high-energy emulsification methods (high-pressure homogenizers, microfluidizers, and ultrasound generators) or by low energy methods (spontaneous emulsification, phase inversion temperature). Depending on the proportion and chemical nature of the components, nanoemulsions can be either water-in-oil (W/O) or oil-in-water (O/W). The application of nanoemulsion formulations for encapsulating, protecting, and improving the delivery of lipophilic functional compounds, with an emphasis on essential oils, has been reported by several research groups [12,13,14,15,16].

Mosquitoes are insects of public health importance due to the nuisance they cause with their bites and the transmission of several diseases to humans, such as malaria, dengue, and West Nile virus [17,18]. Application of larvicides in their breeding sites is one of the main mosquito control measures; however, the repeated use of synthetic insecticides in mosquito control programs has led to mosquito resistance development and adverse effects on nontarget organisms and the environment [19,20,21]. Plant derivatives have been considered as potential alternative agents against mosquito larvae as ecofriendly and more safe substances for nontargets organisms, posing no resistance problems so far [22,23,24]. The efficacy of botanical insecticides is, however, declined by their poor physicochemical stability, high volatility, thermal decomposition, and low water solubility. Nanoformulations have been studied from the perspective of developing suitable delivering systems for the application of essential oils in order to improve their physicochemical characteristics and insecticidal properties against mosquitoes [16,25,26,27]. *Citrus* essential oils and limonene, their dominant component, have been reported for their larvicidal effect against *Culex pipiens molestus* (common house mosquito) and *Aedes albopictus* (Asian tiger mosquito), two mosquito species of great medical importance [28,29,30]. R-(+)-limonene is a biodegradable, nonpolar hydrocarbon with a variety of uses in industrial products, including foods, beverages, cosmetics, and pharmaceuticals. It has also been approved by the US Environmental Protection Agency (EPA) for usage as a natural pesticide and insect repellent.

In the present study, a series of O/W nanoemulsions based on water as the continuous phase and R-(+)-limonene as the dispersed oil phase, polysorbates as surfactants, and alcohols were fabricated using either low or high energy emulsification procedures. Consequently, the nanoemulsions were structurally characterized using different techniques, such as electron paramagnetic resonance (EPR) spectroscopy, dynamic light scattering (DLS), and viscometry. The effect of composition and emulsification process on physical properties (droplet size, polydispersity index, stability, membrane dynamics, viscosity) and in-vivo larvicidal bioactivity of limonene loaded-nanoemulsions on *Ae.albopictus* and *Cx. pipens molestus* were determined.

## 2. Materials and Methods

### 2.1. Chemicals

**Scheme 1:**
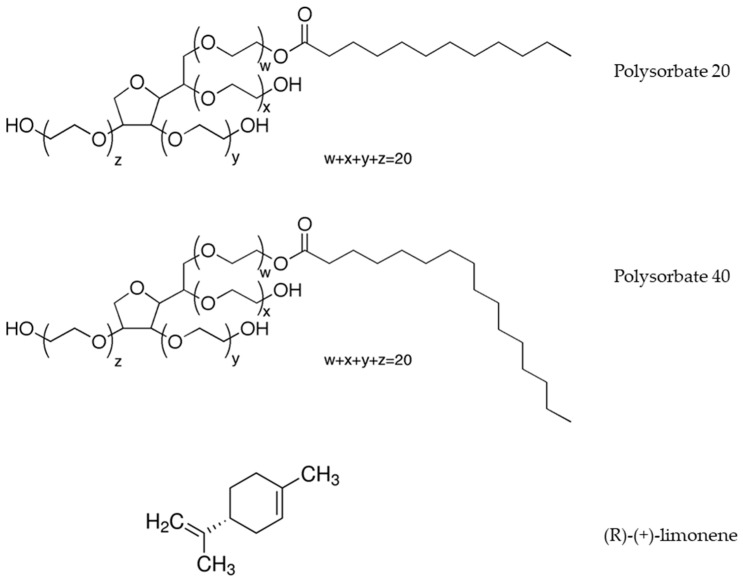
Chemical structure of polysorbates and (R)-(+)-limonene.

(*R*)-(+)-Limonene, 97% purity, was from Alfa Aesar, Karlsruhe, Germany. Ethanol, absolute, analytical reagent was from Fisher Chemical, Loughborough, UK. 1,2-propanediol, 99% purity, was obtained from Sigma-Aldrich, St. Louis MO, USA. Polysorbate 40 was a product of Alfa Aesar, Kandel, Germany. Polysorbate 20 was from Sigma-Aldrich, St. Louis MO, USA. 5-Doxyl stearic acid (5-DSA) was a product of Sigma-Aldrich, Chemie Gmbh Munich, Germany. High-purity water was obtained from a Millipore Milli Q Plus water purification system. Scheme 1 presents the chemical structures of the two polysorbates and (R)-(+)-limonene.

### 2.2. Preparation of Nanoemulsions

Low-energy method: Oil-in-water nanoemulsions (Systems 1–3) were prepared using (*R*)-(+)-limonene, water, polysorbates (Tween 20 or Tween 40), propylene glycol (PG), and ethanol. Initially, mixtures of surfactant, oil, and ethanol were prepared in screw-capped glass vials and allowed to equilibrate in a water bath at 25 °C. The surfactant to oil weight ratio was 4. Then, the mixtures were titrated with the aqueous phase consisting of water and PG in weight ratio 2:1. Each mixture was stirred gently using a Vortex mixer.

High-energy method: Oil-in-water nanoemulsions (System 4) were prepared in a two-step emulsification procedure. Initially, pre-emulsions were obtained by mixing (R)-(+)-limonene, water, propylene glycol, and surfactants at ambient temperature with mechanical stirring. Nanoemulsions were then formulated by passing the pre-emulsions through a Panda PLUS1000 (GEA Niro Soavi, Parma, Italy) high-pressure homogenizer at 700 bar, applying up to 10 recirculation passages. The composition of the nanoemulsions (Systems 1–4) is shown in Table 1.

### 2.3. Viscosity Measurements

Viscosity measurements of the nanoemulsions were performed using a DV-I Prime Digital Viscometer (Brookfield Engineering Laboratories, Middleboro, MA 02346, USA), equipped with a cone spindle (CPA-40Z) (Brookfield Engineering Laboratories, Middleboro, MA 02346, USA). The temperature was kept constant at 25 °C using a water bath. The shear rate was 7.5 s^−1^. Experiments were performed in triplicate, and results were presented as average ± standard deviation (SD).

### 2.4. Dynamic Light Scattering (DLS) Measurements

DLS is a well-established technique for studying colloidal dispersions. The Brownian motion of particles or molecules in suspension causes the laser light to be scattered at different intensities. Analysis of these intensity fluctuations yields the velocity of the Brownian motion and hence the particle size using the Stokes–Einstein equation: RH = kBT/6πηD, where RH is the hydrodynamic radius of the nanoparticle, kB is the Boltzmann constant, T is the temperature in Kelvin, η is the viscosity of the sample, and D is the diffusion coefficient.

Particle size and particle size distribution of the O/W nanoemulsions were measured by dynamic light scattering (DLS) with a Zetasizer Nano ZS (ZEN3600) analyzer (Malvern Panalytical, Malvern, Worcestershire, UK). The analyzer was equipped with a He-Ne laser (633 nm) (Malvern Panalytical, Malvern, Worcestershire, UK) and noninvasive backscatter (NIBS) optics (Malvern Panalytical, Malvern, Worcestershire, UK). Measurements were carried out at the scattering angle of 173° and processed using Malvern Zetasizer Nano software, version 6.32 (Malvern Panalytical, Malvern, Worcestershire, UK), which fits a spherical model of diffusing particles with low polydispersity. The cumulant analysis gives two values, a mean value for the size (intensity mean) and a width parameter known as the polydispersity index (PdI). In the case of concentrated suspensions, multiple scattering becomes the main process, making it impossible to apply a conventional DLS technique without dilution.

### 2.5. EPR Spectroscopy Measurements

Interfacial properties of O/W nanoemulsions were studied by electron paramagnetic resonance (EPR) spectroscopy using the spin-probing technique. EPR is a spectroscopic technique that detects species with unpaired electrons. EPR can yield meaningful structural and dynamic information, often to complement other analytical methods in a wide range of applications. In this study, EPR measurements were performed with a Bruker EMX EPR spectrometer (Bruker Corporation, Billerica, MA, USA) operating at the X-band (9.8 GHz). Samples were contained in quartz flat cells (Wilmad-LabGlass). EPR spectra were recorded at room temperature, with a center field of 0.349 T, scan range 0.01 T, gain of 5.64 × 10^3^, time constant of 10.24 ms, conversion time of 4 ms, modulation amplitude of 0.4 mT, frequency of 9.78 GHz, and power of 2.7 × 10^−1^ mW. Data collection and analysis were performed using the Bruker WinEPR (Bruker Corporation, Billerica, MA, USA) acquisition and processing program.

Initially, a stock solution of the spin probe 16-Doxyl stearic acid (16-DSA) was prepared in absolute ethanol at a concentration of 7.8 mM. Then, 15 μL of the stock solution was added to Eppendorf tubes. After the ethanol was evaporated, 1 mL of the nanoemulsions was added to each Eppendorf tube and kept for 1 day at ambient temperature so that the probe was transferred to the surface of the membranes; 16-DSA concentration in the nanoemulsions was 0.12 mM.

### 2.6. Mosquito Rearing

Mosquito larvae were obtained from laboratory colonies of *Ae. albopictus* and *Cx. pipiens* biotype *molestus*, which were maintained at 25 ± 2 °C, 80% relative humidity, and photoperiod of LD 16:8 h in the laboratory of Benaki Phytopathological Institute, Kifissia, Greece. Adult mosquitoes of each species were kept separately in wood-framed cages (33 × 33 × 33 cm) covered by a 32 × 32 mesh, with easy access to 10% sucrose solution through a cotton wick. *Ae. albopictus* females were chicken blood fed by using the Hemotek membrane feeding system (Hemotek). *Cx. pipiens molestus* females were not blood-fed since *molestus* is an autogenous biotype, i.e., female mosquitoes are able to produce their first egg-batch without a blood meal. Mosquito larvae were reared in tap-water-filled cylindrical enamel pans and were fed ad libitum with powdered fish food (JBL Novo Tom 10% *Artemia*). Cages containing *Cx. pipiens molestus* adults were provided with containers filled with tap water for oviposition, while beakers with water and strips of moistened filter paper were provided as breeding sites for *Ae. albopictus*.

### 2.7. Larvicidal Bioassays

Larval mortality bioassays were carried out according to a modified version of the test method for larval susceptibility recommended by the WHO (2005) and followed in previous studies [30,31]. A series of aqueous solutions with different concentrations of the four systems of oil-in-water nanoemulsions in 100 mL tap water and a stock solution of 10% *w*/*v* (R)-(+)-limonene in dimethyl sulfoxide (DMSO) in 2% (*v*/*v*) aqueous solution of DMSO (98 mL of tap water plus 2 mL of DMSO) were prepared. Twenty late-third- to early-fourth-instar mosquito larvae of each tested mosquito species were exposed to different doses of the tested materials, expressed as mg of (R)-(+)-limonene/L, under laboratory conditions. Gentle shaking to ensure a homogeneous test solution was then performed. Four replicates per dose were made, and treatments with only tap water and 2% water solution of DMSO were included in each bioassay as control.

### 2.8. Data Analysis

The larvicidal effect for lethal bioassays was recorded 24 h after treatment. Data obtained from each dose–larvicidal bioassay (total mortality per milligram of (R)-(+)-limonene per liter of each concentration in water) were subjected to probit analysis in which probit transformed mortality was regressed against log_10_-transformed dose; LC_50_, LC_90_ values, and slopes were generated. All analyses were conducted using the statistical package SPSS 14.0 [32].

## 3. Results and Discussion

### 3.1. Formation, Stability, Viscosity and Membrane Dynamics of O/W Nanoemulsions

A series of oil-in-water (O/W) nanoemulsions were developed, applying either low- or high-energy emulsification techniques depending on their composition. Nanoemulsions are nonequilibrium systems, and their formation requires energy from either mechanical devices or the internal chemical energy of the system. As shown in Table 1, Systems 1 to 3, besides oil, water, and surfactant, also contained a polyol and a short-chain alcohol as cosolvents to achieve spontaneous emulsification of the nanoemulsions [4,33,34]. When the short-chain alcohol (ethanol) was removed (System 4), spontaneous emulsification was not possible and, therefore, mechanical stirring and homogenization under pressure were applied to provide the necessary disruptive forces for the production of nanoscale oil droplets [6,7,35].

Initially, O/W nanoemulsions were developed using (R)-(+)-limonene as the oil phase, mixtures of water and propylene glycol (PG) as the aqueous phase and polysorbates (40 or 20) as surfactants (Table 1, Systems 1–3). In Systems 1 to 3, the surfactant-to-oil weight ratio was 4, and the water-to-PG weight ratio was 2. Ethanol content varied from 2.9% to 8.9% *w*/*w*. For the formulation of System 4, the surfactant-to-oil weight ratio was 2.4, the water-to-PG weight ratio was 2, while there was no addition of ethanol. System 4 was stabilized after mechanical stirring and high-pressure homogenization, applying 10 recirculation passages.

The O/W nanoemulsions were then studied using analytical techniques to elucidate the structural details of the proposed systems in relation to their composition and preparation method. The techniques undertaken were based on scattering, spectroscopy, and viscometry.

Initially, dynamic light scattering (DLS) measurements were carried out to evaluate the size and size distribution of the dispersed oil droplets in the O/W nanoemulsions. Measurements were recorded immediately after preparation and at given time intervals. Table 2 shows the mean droplet size and the polydispersity index of the nanoemulsions (Systems 1–4). As can be observed, Systems 1 and 3 showed similar droplet sizes, 288 ± 12 and 288 ± 17 nm, respectively, but different polydispersity. This finding indicates that the nature of the polysorbate used as a surfactant does not affect the size of the oil droplets as long as the surfactant-to-oil weight ratio remains constant. However, System 1 contained 9.4% *w*/*w* more PG in the aqueous phase, which could possibly affect the PDI of the dispersion. In System 3, the amount of ethanol was kept at 2.9% *w*/*w,* which is the lowest content of all systems. In System 2, limonene, ethanol, and surfactant contents were increased while the total aqueous phase was decreased. In this case, monodispersed (PDI = 0.12 ± 0.01) oil droplets of very low hydrodynamic diameter (16 ± 1 nm) were obtained. Finally, a low oil, low surfactant, and high-water content system was developed in the absence of ethanol (System 4). This system required high disruptive forces to result in oil droplets of 57 ± 1 nm hydrodynamic diameter and PDI 0.233.

**Figure 1:**
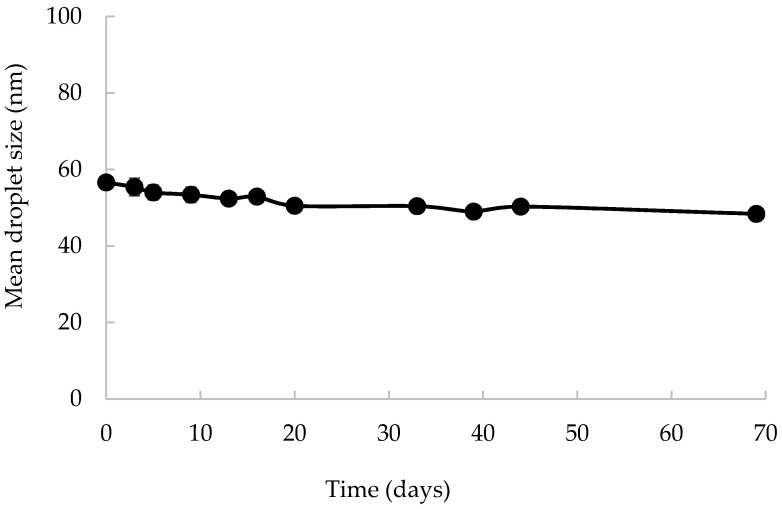
Stability study; mean droplet diameter as a function of time of oil–in–water (O/W) nanoemulsions consisting of 55% *w*/*w* water, 28% *w*/*w* PG, 5% (R)-(+)-limonene, and 12% *w*/*w* polysorbate 40 (System 4).

**Figure 2:**
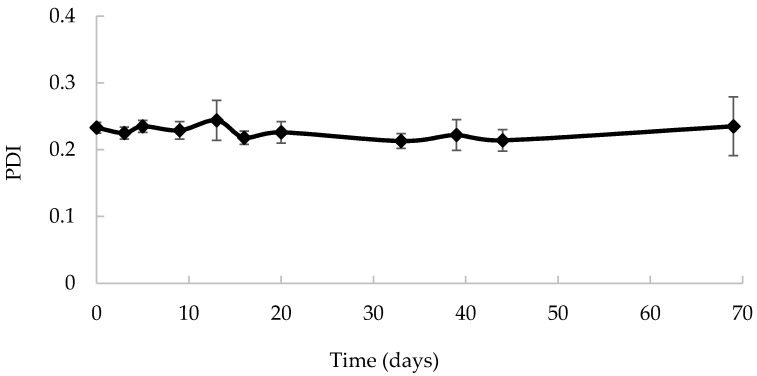
Stability study; polydispersity index as a function of time of O/W nanoemulsions consisting of 55% *w*/*w* water, 28% *w*/*w* PG, 5% (*R*)-(+)-limonene, and 12% *w*/*w* polysorbate 40 (System 4).

The stability of the nanoemulsions was assessed by DLS analysis of droplet size and PDI over time, at a constant temperature. Figure 1 shows the variation of mean droplet diameter with time for the system consisting of 55% *w*/*w* water, 28% *w*/*w* PG, 5% (R)-(+)-limonene, and 12% *w*/*w* polysorbate 40 (System 4) at constant temperature (25 °C). Figure 2 shows the variation of PDI with time for the same system. As can be observed, nanoemulsions were studied for 70 days and remained stable throughout this time. In addition, no phase separation or other visual changes occurred. The same behavior was also observed for Systems 1 to 3, which contained ethanol and were formulated with a low-energy technique.

The viscosity of all prepared nanoemulsions was measured and found to be <140 centipoise (cP) (Table 2). This finding is consistent with one of the basic characteristics of nanoemulsions, which is low viscosity. System 4 showed the lowest viscosity (3.7 ± 0.6 cP), which is related to the high percentage of water (55% *w*/*w*) and the low concentration of Tween 40 (12% *w*/*w*). The viscosity of System 2 was also very low, mainly due to the presence of the less viscous surfactant Tween 20 and the increased concentration of ethanol (8.9% *w*/*w*).

To summarize, the emulsification procedure, viscosity, size, and size distribution of the dispersed oil droplets in the O/W nanoemulsions were affected by the composition, with emphasis on the presence of ethanol. Stability during storage was not affected by the composition for a period of at least 70 days.

To extend these findings, EPR spectroscopy was applied to study the interfacial properties of the surfactants’ monolayer in the nanoemulsions. For this purpose, 16-DSA, a spin-labeled fatty acid analog consisting of stearic acid and an N–O^.^ moiety attached to the C-16 position of the hydrocarbon chain, was used as a spin probe. Due to its amphiphilic nature, 16-DSA was localized at the oil/water interface interacting with the surfactant molecules. In the present study, the EPR spectra of 16-DSA were analyzed to provide information regarding mobility, order, and polarity across the surfactant’s monolayer. For this purpose, rotational correlation time (τ_R_), order parameter (S), and isotropic hyperfine splitting constant (α_N_) were calculated from the EPR spectra, as reported elsewhere, and the results are shown in Table 3 [12,34]. In all systems, rotational correlation times (τ_R_) were at the fast motion region (τ_R_ < 3 ns), ranging from 0.31 to 0.42 ns, indicating a fluid interface of low local viscosity. In addition, the order parameter (S) was practically the same in all nanoemulsions (0.03), showing the existence of surfactants’ layers with a low degree of order. Finally, local polarity, as expressed by the hyperfine splitting constant (α_N_), was also similar in all systems.

Overall, EPR results show that the composition and preparation method of the four nanoemulsions did not affect the fluidity, rigidity, and polarity of the surfactants’ monolayer at least from the depth where the doxyl-ring of the 16-DSA is located, namely, the 16th carbon atom. In this position, the doxyl-ring bearing the free electron is closer to the oil phase and far from the polar head groups and the aqueous phase.

### 3.2. Insecticidal Effect

Larval toxicities of the four limonene nanoemulsions and pure limonene against *Ae. albopictus* and *Cx. pipiens molestus* are presented in Table 4. All tested materials showed increasing mortality, with increasing concentration against the third to fourth larval stages of mosquitoes, while no mortality was recorded in the untreated controls. The dose–response test against *Ae. albopictus* revealed a higher larvicidal effect (significantly lower LC_50_ values) of limonene-based nanoemulsions in comparison with limonene dissolved in an organic solvent. Similarly, the performance of limonene on *Cx. pipiens molestus* larvae when formulated in nanoemulsions was either equal to (Systems 1, 2, and 4) or better (System 3) than in the simple solution. The improved bioefficacy of limonene in nanoformulations may be attributed to benefits of nanomaterial-based formulations, such as the small size of droplets that amplifies the penetration and transmission of the insecticidal substance through the exoskeleton in the larval body, the stability of tested nanosystems having the advantage of protecting the active substance against mechanisms of inactivation and degradation, or the better water solubility [26,27,36,37]. Larvicidal activity of limonene nanoformulations against mosquitoes was also demonstrated by [38]. They produced nanoemulsions based on the essential oil (EO) of *Baccharis reticulata*, as well as D-limonene, its major component (25%). Limonene nanoemulsion, having mean droplet size 136 nm and polydispersity index 0.7, caused the mortality of *Ae. aegypti* larvae with an LC_50_ value of 91.25 μg/mL after 24 h of exposure. The larvae treated with D-limonene nanoemulsion presented a fragile appearance and a wrinkled body surface, showing alterations on head, thorax, siphon, and on the cuticle of the abdomen. The evaluation of a nanoemulsion containing the EO of *Cirtus sinensis* resulted in 50% mortality (LC_50_) of *Culex pipiens* larvae at 27.4 ppm, which is in accordance to our limonene nanoformulations [39].

Laboratory bioassays showed a superior insecticidal effect of System 3 (lowest LC_50_ values), which is a limonene nanoemulsion consisting of polysorbate 20 (39.5% *w*/*w*), water (32.6% *w*/*w*), propylene glycol (16.3% *w*/*w*), and ethanol (2.9% *w*/*w*). Interestingly, the fact that System 3 was more viscous than other systems due to the high percentage of surfactant and low ethanol content (Table 2) did not affect the ability of the nanoemulsions to effectively release limonene and exhibit enhanced insecticidal activity. As it has been reported in the literature that the viscosity of the colloidal structures used for the delivery of active compounds in general plays an important role in the effective release of the encapsulated compounds [40,41]. The emulsification technique, which is related to the size and stability of the nanoemulsions, does not seem to affect the activity of the formulations. EPR results and rotational correlation times (τ_R_) showed that the surfactants’ monolayer was more fluid when System 3 was considered. In general, this kind of structural information is important for emulsion-based delivery systems because the fluidity of the interfacial layer is related to the performance of the formulation and the delivery of the encapsulated compound [42,43].

## 4. Conclusions

In the current study, we produced four R-(+)-limonene based oil-in-water nanoemulsions employing low- (Systems 1–3) and high- energy (System 4) methods, and we studied their physicochemical characteristics such as viscosity, stability, mean droplets size, polydispersity index, and rotational correlation time using appropriate methods. Bioactivity of the tested nanoformulations was tested in the laboratory against mosquito larvae of *Ae. albopictus* and *Cx. pipiens molestus.* In the bioassays, a simple solution of limonene in organic solvent was included as a positive control. Dose–response tests showed that all tested nanoformulations enhanced the efficacy of limonene against *Ae. albopictus* larvae, while similar or higher toxicity was observed against *Cx. pipiens molestus* in comparison with pure limonene, depending on the applied nanosystem. In conclusion, limonene was successfully formulated in nanoemulsions with sufficient toxic properties against mosquito larvae, having the potential to be applied in integrated mosquito control programs.

## Figures and Tables

**Table 1:** Composition of the oil-in-water (O/W) nanoemulsions.

	System			
Ingredients (% *w*/*w*)	1	2	3	4
(R)-(+)-Limonene	3.8	8.9	8.7	5
Polysorbate 40	15.3	-	-	12
Polysorbate 20	-	36.9	39.5	_
Water	51.4	30.2	32.6	55
Propylene Glycol	25.7	15.1	16.3	28
Ethanol	3.8	8.9	2.9	-

**Table 2:** Characteristics of the O/W nanoemulsions. Mean droplet diameter, polydispersity index (PdI), and viscosity were calculated immediately after preparation. All experiments were performed in triplicate, and results were presented as average ± standard deviation (SD).

System	Size (nm)	PdI	Viscosity (cP)
1	288 ± 12	0.7 ± 0.03	102.7 ± 5.7
2	16 ± 1	0.12 ± 0.01	9.2 ± 1.3
3	288 ± 17	0.16 ± 0.03	137.4 ± 27.5
4	57 ± 1	0.23 ± 0.01	3.7 ± 0.6

**Table 3:** Rotational correlation time (τ_R_), isotropic hyperfine splitting constant (α′_ο_), and order parameter (*S*) of 16-DSA in the O/W nanoemulsions.

System	τ_R_ (ns)	S	α′_ο_ (×10^−4^ T)
1	0.42 ± 0.01	0.03 ± 0.00	14.79 ± 0.01
2	0.38 ± 0.08	0.03 ± 0.00	14.76 ± 0.3
3	0.31 ± 0.0	0.03 ± 0.00	14.74 ± 0.03
4	0.40 ± 0.01	0.03 ± 0.00	14.73 ± 0.02

**Table 4:** LC_50_ and LC_90_ values of four limonene-based nanoemulsions (Systems 1–4) and limonene dissolved in an organic solvent (DMSO) against the third- to fourth-instar larvae of *Ae. albopictus* and *Cx. pipiens molestus* after 24 h exposure (SE: standard error; d.f.: degrees of freedom).

Type of Limonene Formulation Applied	Slope (±SE)	LC_50_ (95% CL) ^a^	LC_90_ (95% CL) ^a^	*x^2^*	d.f.
*Aedes albopictus*					
System 1	7.3 ± 0.7	30.6 (28.8–32.4)	45.9 (42.8–50.5)	18.260	13
System 2	5.9 ± 0.6	27.1 (23.8–30.6)	44.5 (38.2–57.6)	28.585 ^b^	13
System 3	3.9 ± 0.3	20.1 (18.2–22.1)	42.7 (37.2–51.7)	47.560 ^b^	28
System 4	5.5 ± 0.5	29.6 (27.2–31.9)	50.8 (45.3–60.7)	42.075	23
R-(+)-limonene	9.7 ± 1.1	36.0 (32.6–39.5)	48.8 (43.6–60.9)	26.174 ^b^	10
*Cx. pipiens molestus*					
System 1	6.4 ± 0.6	34.6 (30.7–38.1)	54.7 (48.5–66.9)	41.323 ^b^	16
System 2	4.1 ± 0.3	33.7 (29.9–37.5)	69.5 (61.5–80.7)	23.062	15
System 3	4.5 ± 0.3	23.9 (21.9–26.1)	46.4 (40.7–55.5)	46.229 ^b^	28
System 4	5.8 ± 0.5	30.9 (28.1–34.1)	51.9 (44.7–65.5)	42.075 ^b^	23
R-(+)-limonene	4.9 ± 0.5	32.2 (27.6–36.7)	58.2 (50.6–72.4)	22.705 ^b^	13

^a^ LC values are expressed in mg (R)-(+)-limonene L^−1^. and they are considered significantly different when 95% confidence limits (CLs) fail to overlap. ^b^ Since goodness–of–fit test is significant (*p* < 0.05), a heterogeneity factor is used in the calculation of CLs.
